# Enhanced or Reduced Fetal Growth Induced by Embryo Transfer into Smaller or Larger Breeds Alters Post-Natal Growth and Metabolism in Pre-Weaning Horses

**DOI:** 10.1371/journal.pone.0102044

**Published:** 2014-07-09

**Authors:** Pauline Peugnet, Laurence Wimel, Guy Duchamp, Charlotte Sandersen, Sylvaine Camous, Daniel Guillaume, Michèle Dahirel, Cédric Dubois, Luc Jouneau, Fabrice Reigner, Valérie Berthelot, Stéphane Chaffaux, Anne Tarrade, Didier Serteyn, Pascale Chavatte-Palmer

**Affiliations:** 1 INRA, UMR1198 Biologie du Développement et Reproduction, Jouy en Josas, France; 2 ENVA, Maisons Alfort, France; 3 IFCE, Station Expérimentale de la Valade, Chamberet, France; 4 INRA, UE1293, Nouzilly, France; 5 Clinique équine, Faculté de Médecine Vétérinaire, CORD, Université de Liège, Liège, Belgique; 6 INRA, UMR85, Physiologie de la Reproduction et Comportements, Nouzilly, France; 7 CNRS, UMR7247, Nouzilly, France; 8 Université François Rabelais de Tours, Tours, France; 9 IFCE, Nouzilly, France; 10 INRA, UMR791 Modélisation Systémique Appliquée aux Ruminants, Paris, France; 11 AgroParis Tech, Paris, France; University of Tasmania, Australia

## Abstract

In equids, placentation is diffuse and nutrient supply to the fetus is determined by uterine size. This correlates with maternal size and affects intra-uterine development and subsequent post-natal growth, as well as insulin sensitivity in the newborn. Long-term effects remain to be described. In this study, fetal growth was enhanced or restricted through ET using pony (P), saddlebred (S) and draft (D) horses. Control P-P (n = 21) and S-S (n = 28) pregnancies were obtained by AI. Enhanced and restricted pregnancies were obtained by transferring P or S embryos into D mares (P-D, n = 6 and S-D, n = 8) or S embryos into P mares (S-P, n = 6), respectively. Control and experimental foals were raised by their dams and recipient mothers, respectively. Weight gain, growth hormones and glucose homeostasis were investigated in the foals from birth to weaning. Fetal growth was enhanced in P-D and these foals remained consistently heavier, with reduced T_3_ concentrations until weaning compared to P-P. P-D had lower fasting glucose from days 30 to 200 and higher insulin secretion than P-P after IVGTT on day 3. Euglycemic clamps in the immediate post-weaning period revealed no difference in insulin sensitivity between P-D and P-P. Fetal growth was restricted in S-P and these foals remained consistently lighter until weaning compared to S-D, with elevated T_3_ concentrations in the newborn compared to S-S. S-P exhibited higher fasting glycemia than S-S and S-D from days 30 to 200. They had higher maximum increment in plasma glucose than S-D after IVGTT on day 3 and clamps on day 200 demonstrated higher insulin sensitivity compared to S-D. Neither the restricted nor the enhanced fetal environment affected IGF-1 concentrations. Thus, enhanced and restricted fetal and post-natal environments had combined effects that persisted until weaning. They induced different adaptive responses in post-natal glucose metabolism: an early insulin-resistance was induced in enhanced P-D, while S-P developed increased insulin sensitivity.

## Introduction

Epidemiological studies in humans have linked early-life events with a range of pathologies in adulthood. The first evidence of this was provided by the Hertfordshire's cohort in which people who had a small birth weight (reflecting suboptimal fetal development) were at greater risk of developing coronary heart disease, hypertension or type II diabetes in later life [1]–[3]. Maternal nutrition was pointed out as the primary factor affecting fetal development: in investigations of individuals who were exposed *in utero* to the Dutch Famine during World War II. It was shown that they were prone to a higher risk of developing obesity, glucose intolerance, hypertension or cardiovascular diseases in adult life [4], [5]. Rapid post-natal catch-up growth was also shown to increase the risk of later obesity as a result of a mismatch between the restricted *in utero* conditions to which the fetus had adapted and post-natal abundance [6]. In contrast, excess birthweight also leads to adverse programming, with a U-shaped curve for increased risks [7].

Experiments aimed at compromising fetal and neonatal development in animal models have confirmed that *in utero* and neonatal developmental conditions impact an individual's risk of developing metabolic diseases as an adult [8]. Indeed, intra-uterine growth retardation (IUGR) may lead to a post-natal increase in blood pressure and glucose intolerance [9] and may affect pancreatic islet function [10], the renin-angiotensin system [11] and the hypothalamic-pituitary-adrenal axis [12], depending on the individual's genotype and sex [13], [14], as well as on the timing and intensity of the perturbation [15].

In production animals, the Developmental Origins of Health and Disease (DOHaD) are of interest for their role in programming characteristics linked to commercial benefits, such as offspring survival, growth rate, body composition, fleece, milk and meat qualities and reproductive function [16], [17]. Alterations in the fetal environment could also limit future health and athletic performance of the horse [18]. IUGR in equids has been reported to induce various detrimental effects in newborn foals and older horses, affecting the pulmonary microstructure balance, the respiratory function efficiency, the development of neuropathies or hyperlipidemia, as well as muscle and skeleton development and function [19]. Recently, an epidemiological study performed in Belgium underlined the detrimental effect of feeding pregnant mares with concentrates on the post-natal development of osteochondrosis lesions in their offspring [20]. These factors moderate the importance of genetics and post-natal life environment, highlighting the role of early developmental events in later athletic capacities in the horse. Early impacts on energy homeostasis in horses, although still unclear, are also of strong interest, since insulin resistance is involved in various pathologies of the adult horse such as Cushing's syndrome, laminitis, type II diabetes, hyperlipidemia, endotoxemia or osteochondrosis, as well as the equine metabolic syndrome [21], [22]. Moreover, obesity in adult mares has been linked to reduced reproductive performance [23].

In horses, placentation is epitheliochorial and occurs over the entire surface of the endometrium. Thus, the nutritional supply to the fetus, which depends on the contact surface between the placenta and the endometrium, is governed by the size of the uterus and therefore by the mare's size. Based on this observation, the impact of early life events on intra-uterine and post-natal development of the foals was demonstrated using artificial insemination to cross large Shire horses with small Shetland ponies [24]. More recently, Allen and his colleagues used embryo transfers between small and large breeds of *equidae* (ponies and thoroughbreds) as a model for fetal programming, restricting or enhancing fetal growth by transferring thoroughbred embryos into pony mares and pony embryos into thoroughbred mares, respectively. Fetal development was related to maternal size, with the gross placental area, weight and microcotyledonary density being the primary operative control mechanisms [25]. Increased or restricted post-natal growth of foals born to between-breeds embryo transfers were also associated with altered blood pressure and response of catecholamine to acute stress [26] and altered pancreatic β cell function [27] in the immediate neonatal period (first week after birth).

The long term effects of these transfers on daily weight gain, glucose homeostasis and endocrine factors involved in growth remain unknown. Moreover, in embryo transfer practice, recipient mares are used which may not be the same size and breed as the embryo. This may lead to physiological adaptations that could affect offspring's pre- and post-natal development. The objectives of this work were to revisit Allen *et al*'s study and explore long term metabolic effects on offspring. Fetal growth was increased by transferring pony and saddlebred embryos into draft mares and restricted by transferring saddlebred embryos into pony mares. Foals were monitored from birth to weaning for weight gain, glucose homeostasis and endocrine factors involved in both growth and energy regulation.

## Materials and Methods

The animal studies were approved by the local animal care and use committee (“Comité des Utilisateurs de la Station Expérimentale de Chamberet”) and received ethical approval from the local ethics committee (“Comité Régional d'Ethique pour l'Expérimentation Animale du Limousin”) under protocol number 5-2013-5.

**Figure 1:**
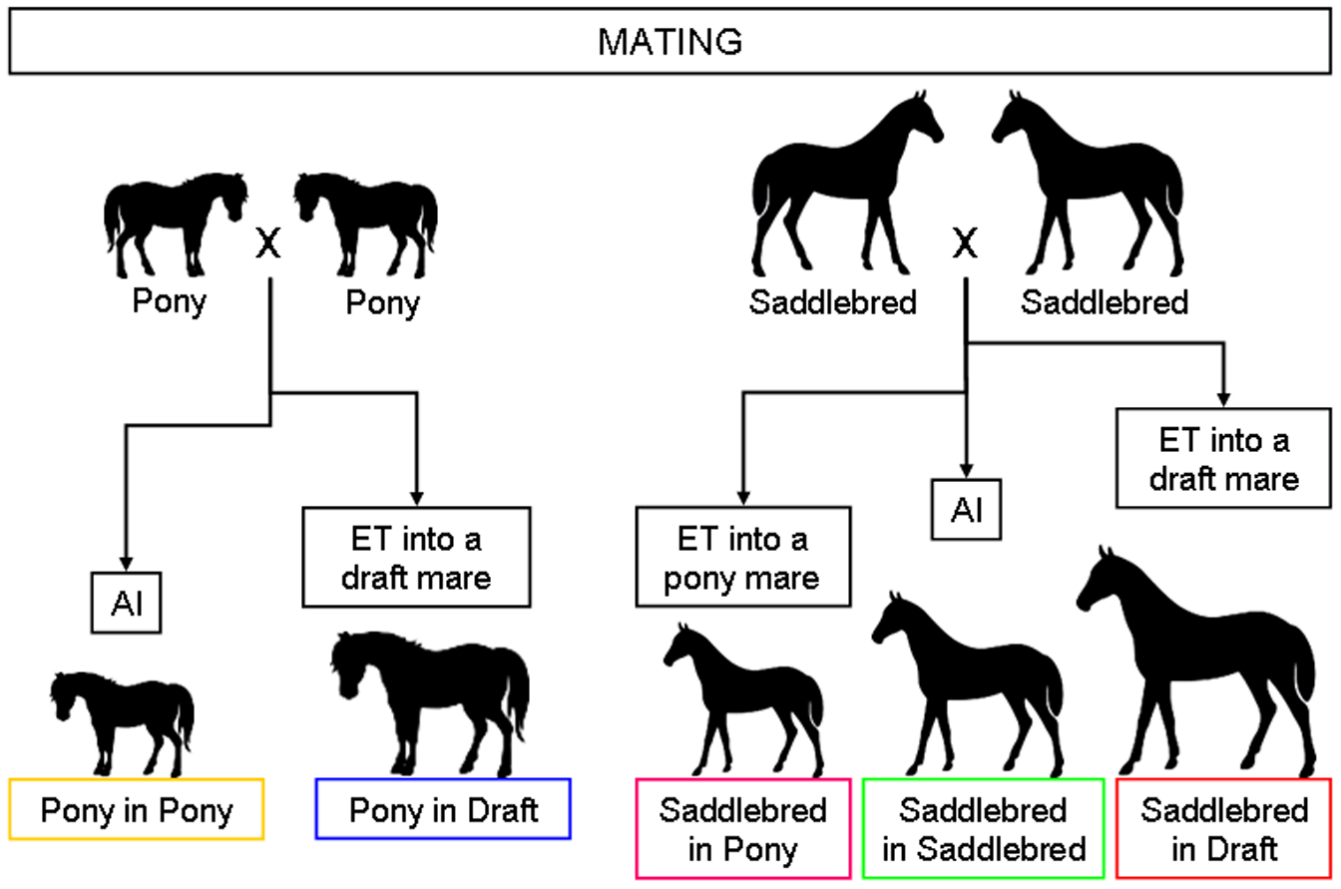
Establishment of control and experimental pregnancies by artificial insemination (AI) and embryo transfer (ET), respectively.

The study was conducted over 2 successive breeding seasons (foaling in 2011 and 2012). Pony mares (n = 27) were located at the Institut National de la Recherche Agronomique (INRA) experimental farm in Nouzilly, France (farm 1, altitude 120 m). Pony embryos (n = 61) were produced in the same location. Saddlebred (n = 28) and draft (n = 14) mares were located at the Institut Français du Cheval et de l'Equitation (IFCE) experimental farm in Chamberet, France (farm 2, altitude 470 m). Saddlebred embryos (n = 48) were produced in the same location. Median mares' age was 6.9 years (range 3 to 19 years) and the herd included primiparous or multiparous mares (up to 10 gestations). With a median age of 4 years [3]–[5], draft mares were significantly younger than pony and saddlebred mares (9 years [5]–[10] and 7 years [4]–[13], respectively; p<0.000 with the Kruskal-Wallis test). With a median parity of 2 foals [1]–[4], saddlebred mares had significantly higher parity than pony and draft mares (1 foal [1]–[2] for both breeds, p = 0.007 with the Kruskal-Wallis test). For the whole experiment, 1 pony stallion and 2 saddlebred stallions of the same breed and size (1.6 m at withers) were used. The experimental protocol used to produce foals is described in Figure 1.

### Control pregnancies: within-breed artificial insemination (AI)

The number of animals and their use over the two experimental years are described in Table 1.

**Table 1:** Number of recipient and control mares and foals with sex ratio within the five groups.

		P-P	P-D	S-P	S-S	S-D
**Number of mares and foals**	**2011**	10	5	2	18	8
	**2012**	11	1	4	10	0
	**Total**	21	6	6	28	8
**Number of females/number of males**		12/9	4/2	2/4	16/12	6/2

All mares were pregnant and delivered one foal, so mare numbers are the same as foal numbers.

(P-P: Pony in Pony, P-D: Pony in Draft, S-P: Saddlebred in Pony, S-S: Saddlebred in Saddlebred, S-D: Saddlebred in Draft).

Pony-in-Pony (P-P) and Saddlebred-in-Saddlebred (S-S) pregnancies were obtained by artificial insemination using semen from 1 pony and 2 saddlebred stallions, respectively. Follicular growth and ovulation were monitored by transrectal ultrasonography in order to determine the timing of insemination. Pregnancy was checked 14 days after ovulation by transrectal ultrasonography.

### Experimental pregnancies: between-breed embryo transfer (ET)

Pony-in-Draft (P-D), Saddlebred-in-Pony (S-P) and Saddlebred-in-Draft (S-D) pregnancies were obtained as described below. Embryo donors (pony and saddlebred mares) and recipients (pony and draft mares) cycles were synchronized with an intramuscular injection of prostaglandin analogue (0.125 mg Estrumate (MSD Santé Animale, Beaurouzé, France) for pony mares; 7.5 mg Prosolvin (Virbac) for saddlebred and draft mares) to induce luteolysis. Donors and recipients were subsequently given 15 mg crude equine gonadotropin (pony mares) or 750 IU Chorulon (MSD Santé Animale) (saddlebred and draft mares) intravenously to induce ovulation and donors were artificially inseminated. The donors' uteri were flushed 3 times with one liter of Ringer lactate solution 7 days after ovulation. Recovered embryos were washed 10 times in Emcare Holding solution (ICP bio), transported in an Equitainer (Hamilton Research) to the other experimental farm (3–4 hours) and immediately transferred non-surgically into synchronized recipients 5 to 7 days post-ovulation. Pregnancy was diagnosed by ultrasound 7 days after transfer (corresponding to a 14-day pregnancy).

### Nutrition and general care

From the day of ovulation, grazing was available 24 h/day with free access to water and mineral salts for all pregnant mares. From the 5^th^ gestational month (November, fall), they were housed in boxes and fed a diet based on straw and hay complemented with concentrates (soybean or commercial pellets (Eperon, Tellus Nutrition Animale, France) on farm 1 and either homemade pellets containing barley, soybean cake, molasses and minerals and vitamins, or moha hay on farm 2) with free access to water and mineral salts. The quality of feedstuff was measured for each new batch and is detailed in Table S1.

All foals were born during spring and summer (range April 6^th^–August 13^th^) with the majority (>75%) being born in May and June. Mares and foals returned to grazing 10 and 3 days after foaling, respectively on farms 1 and 2. At each farm, fillies and colts were raised in one group in the same pasture until weaning at 180 days of age. From weaning, foals were housed in open barns and fed a diet based on straw and hay complemented with concentrates commercial pellets (Eperon, Tellus Nutrition Animale) on farm 1 and homemade pellets containing barley, soybean cake, molasses and minerals and vitamins on farm 2 (Table S1). Horses had free access to water on both farms and free access to mineral salts on farm 1. The foals were vaccinated and dewormed as for standard care.

### Body condition and weight gain monitoring and blood sampling in mares and foals

Mares were weighed and scored for body condition (Body Condition Score – BCS - scale 1 to 5 [28]) every 2 months from the 5^th^ gestational month (when mares were housed in boxes), on day 1 post foaling and then monthly. The same person performed all BCS within each farm. Blood samples were collected on EDTA from the jugular vein at the same time of the day (9–10 AM) to measure plasma concentrations of non esterified fatty acids (NEFA) and leptin every 2 months from the 5^th^ gestational month, on day 1 post foaling and then every two months. Due to a technical problem, samples could not be obtained from all mares at 5 and 6 months, and thus some comparisons could not be performed at these stages. Foals were weighed on the morning following birth, at 2 weeks of age and then monthly after foaling. Blood samples were collected on EDTA from the jugular vein before first suckling, at 3 and 30 days of age after 4 h fasting, then monthly until 180 days of age after 6 h fasting and at 200 days of age after overnight fasting. Fasting glucose was measured at the same time at 3, 30, 90, 140, 180 and 200 days of age using an automated analyzer (Medisense Optium Xceed, Abbott, Illinois, USA).

### Intravenous glucose tolerance test (IVGTT) in foals at 3 days of age

#### Experimental procedure

Foals were muzzled to prevent them from suckling 4 h before the procedure. Just before starting the test, a catheter (14G, Introcan-W Certo, BBraun, Melsungen, Germany) with an extension tube was placed in the left jugular vein. Foals were infused intravenously with glucose (0.25 g/kg, 30% glucose, BBraun) over 1 min through this catheter. Blood samples were collected on EDTA from the right jugular vein at −1 min and 1, 3, 5, 7, 9, 12, 15, 30 and 60 min after glucose infusion for immediate measurement of glycemia using an automated analyzer (Medisense Optium Xceed). Blood samples were centrifuged at 3,500 g for 10 min and plasma was separated and stored frozen at −20°C until insulin assay.

#### Calculations

The areas under the glucose and insulin response curves (AUC) were calculated with the trapezoidal method, reflecting the integrated plasma concentration after glucose administration from −1 to 60 min above the pre-infusion baseline for all positive values. Maximum plasma glucose and insulin increments at each time point and AUC for insulin and glucose were compared.

### Hyperinsulinemic euglycemic clamp in foals at 200 days of age

#### Experimental procedure

The procedure reported below is adapted from previous published work [29]. One catheter (14G, Introcan-W Certo, BBraun) was placed in each jugular vein after feed had been withheld for 12 h. One of the catheters was used for infusion of 30% glucose and insulin (recombinant human insulin, Actrapid, 100 IU/mL, Novo Nordisk A/S, Bagsvaerd, Denmark) and the other one for blood sampling. A priming dose of 45 mU of insulin/kg diluted in 0.9% sodium chloride (Aguettant, Lyon, France) was given intravenously within 2.5 min to induce hyperinsulinemia. Immediately after the administration of the insulin priming dose, insulin infusion was started with a constant infusion rate of 6 mU/kg/min. Glucose infusion was started simultaneously with an infusion rate of 8.6 µmol/kg/min. During the insulin and glucose infusions, glycemia was measured every 10 min using the same automated analyzer as described above. The glucose infusion rate was adjusted when the preceding glycemia value differed from the euglycemic concentration (range 4.4 to 6.7 mmol/L) until a steady state was obtained. The steady state was maintained for at least 40 min and 3 blood samples were collected on EDTA (at the beginning, middle and end of the steady state), centrifuged at 3,500 g for 10 min and plasma was separated and stored at −20°C until insulin assay.

#### Calculations

The glucose metabolism rate was calculated as follows: M (mmol/kg/min) = INF (mmol/kg/min)−SC (mmol/kg/min), where M is the glucose metabolism rate, INF is the glucose infusion rate and SC is the space correction factor. The SC was calculated as follows: SC (mmol/kg/min) = (G2−G1)×0.019, where G1 and G2 are the glycemia values before and after each 10-min period.

### Plasma leptin analysis

Mare's plasma leptin concentrations were measured in duplicate with a homologous double-antibody RIA developed in our laboratory [30] with some modifications. The primary antibody was obtained from goats immunized against recombinant equine leptin (a gift from A. Gertler, the Hebrew University, Rehovot, Israel). Standards (0.75 to 40 ng recombinant equine leptin/mL) and samples (aliquots of 100 µL) diluted to 350 µL in PABET (Protein Assay Buffer with EDTA and Tween 20) pH 7.2 were incubated for 24 h at room temperature (RT) with equine leptin antiserum (50 µL at 1∶3,000 initial dilution). After the initial incubation, 100 µL of ^125^I- equine leptin (diluted in the same buffer without EDTA) were added to each tube and the incubation continued at RT. After approximately 24 h the tubes were placed at 4°C until the end of the assay the next day. The antigen-antibody complex was precipitated following a 35-min RT incubation with 100 µL of a rabbit anti-goat antiserum and by centrifugation at 2,700 g for 35 min. The limit of detection was 1.0 ng/ml. Intra- and inter-assay coefficients of variation were less than 10% and 10–13%, respectively.

### Plasma NEFA analysis

Mare plasma NEFA concentrations were measured in duplicate with an enzymatic-colorimetric method using a Cobas Mira-analyzer (Roche, Mannheim, Germany) with a commercial kit for NEFA (NEFA-HR(2), Wako Chemical GmbH, Neuss, Germany). The minimum level of detection was 10 µmol/L. Intra- and inter-assay coefficients of variation were 2.7% and 4.5%, respectively.

### Plasma IGF-1, T_3_ and T_4_ analyses

Foal fasting plasma IGF-1, T_3_ and T_4_ concentrations were measured in duplicate with commercial RIA kits (IGF-1-RIACT, OCPE07-T3 and OCPG07-T4, CISbio International, Gif sur Yvette, France) validated for use in horses. The minimum levels of detection were 1.0 ng/mL, 0.1 ng/mL and 2.5 ng/mL, respectively for plasma IGF-1, T_3_ and T_4_. The intra- and inter-assay coefficients of variation were 3.5% and 6.0% for plasma IGF-1, 7.8% and 8.2% for plasma T**_3_** and 4.7% and 8.0% for plasma T**_4_**, respectively.

### Plasma insulin analysis

Foal fasting and post-bolus plasma insulin concentrations were measured in duplicate with a double antibody RIA as previously described. The minimum level of detection was 0.1 pg/mL and the intra-assay coefficients of variation were 7.2% and 5.8%, respectively.

### Statistical analysis

All results are expressed as median [quartile 1 - quartile 3] and are presented as curves (median and interquartile range) or boxplots (minimum to maximum). Most values are presented in Table S2 for mare parameters and in Table S3 for foal parameters. Statistical analysis were carried out using R software (www.r-project.org/, version i386 2.15.2).

Analyses on mares were performed in two stages: 1) the effect of the maternal breed and 2) the effect of embryo transfer were studied. Mare parameters (body weight, body score, NEFA and leptin) were analyzed at each time point using the coin plug-in for Rcmdr [31] with the Kruskal-Wallis test followed by the NDWD *post-hoc* test for question 1 (pony *vs* saddlebred *vs* draft mares) and with the Mann-Whitney rank sum test for question 2 (P-P *vs* S-P and P-D *vs* S-D).

Three factors of variation were successively analyzed in foals: 1) breed effect (pony *vs* saddlebred controls), 2) effect of increased fetal growth in ponies and 3) either restricted or increased fetal growth in saddlebred foals. Non repeated measures were analyzed using the coin plug-in for Rcmdr with the Mann-Whitney rank sum test for question 1 (P-P *vs* S-S) and 2 (P-D *vs* P-P) and with the Kruskal-Wallis test followed by the NDWD *post-hoc* test for question 3 (S-P *vs* S-S *vs* S-D) [32]. Repeated measures were first analyzed using the F1 LD F1 model of the nparLD function to calculate an ANOVA-type statistic followed by paired comparison to answer the question of a group effect for each pair of groups [32]. Data were then analyzed at each time point with the Mann-Whitney rank sum test for question 1 (P-P *vs* S-S) and 2 (P-D *vs* P-P) and with the Kruskal-Wallis test followed by the NDWD *post-hoc* test for question 3 (S-P *vs* S-S *vs* S-D). Sex-associated differences were also studied within each control groups with the Mann-Whitney rank sum test (males *vs* females).

Data were considered statistically significant for p<0.05. P-values below 0.0005 are indicated as p<0.000.

## Results

### Pregnancy and parturition outcomes

The number of foals and sex ratio within groups are shown in Table 1. Over the two breeding seasons (2011 and 2012), 21 P-P and 28 S-S control foals were born and 6 P-D, 6 S-P and 8 S-D experimental foals were obtained. All the mares delivered spontaneously at term. All the foals were healthy but 3 S-P foals (out of 6) exhibited signs of prematurity/dysmaturity [33] and needed assistance to stand and suckle for the first days after birth. Consequently, 3 S-P foals and their pony dams were not allowed to go to pasture before 30 days of age as their counterparts did. Six S-S foals died: 2 fillies and 3 colts died from diarrhea and 1 colt was euthanized because of septic arthritis in the first week after birth. One S-D foal was rejected by its draft dam and was bottle-fed until weaning. Data collected for these foals were however not discarded from further analysis because they were not identified as outliers for any studied parameter. All the foals were weaned at 180 days of age. Values for mares and foals parameters are shown in Table S2 and Table S3.

### Mares during gestation and lactation

#### Body weight

**Figure 2:**
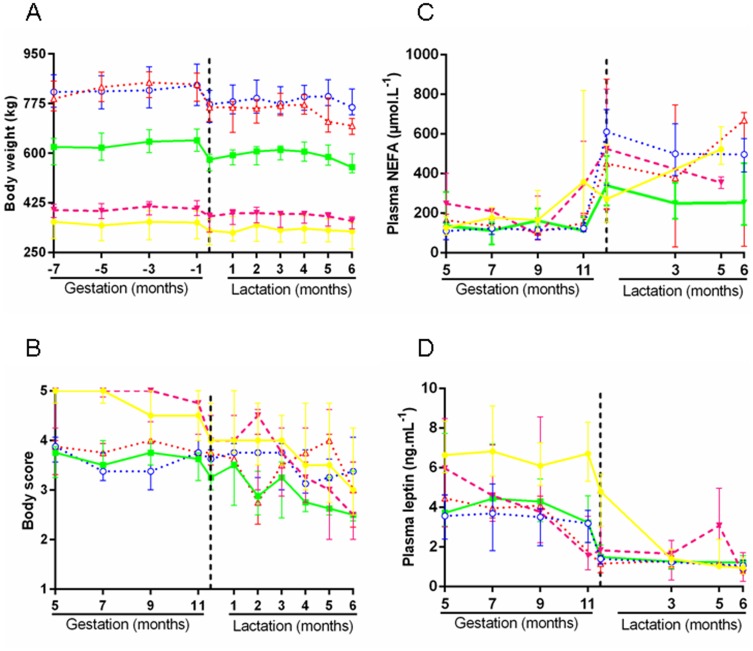
Mares' parameters from the 5^th^ gestational month to weaning in the five groups. **A:** body weight. **B:** body scores. **C:** plasma NEFA. **D:** plasma leptin. (P-P: Pony in Pony (•), P-D: Pony in Draft (○), S-P: Saddlebred in Pony (▾), S-S: Saddlebred in Saddlebred (▪), S-D: Saddlebred in Draft (Δ)). Curves are presented as medians and interquartile ranges.

Mare body weights remained constant throughout gestation as illustrated in Figure 2A, with marked differences between breeds (median body weight 375.0 kg [319.5–406.3], 635.5 kg [589.2–671.8], and 827.7 kg [780.0–874.0] in pony, saddlebred and draft mares, respectively). Body weight decreased in all mares after foaling (−9.3%, −10.5% and −1.3% in pony, saddlebred and draft mares, respectively, p<0.000). Body weights remained stable thereafter until around 2 months *postpartum* when they gradually decreased until weaning at 6 months.

At the time of ET into ponies, when several recipients were available, the larger pony mare was selected as recipient in order to reduce putative problems linked to the foal's size at parturition. Indeed, pony mares pregnant with a saddlebred fetus were significantly heavier with larger withers' height compared to pony mares carrying pony pregnancies (body weight 402.5 kg [389.5–425.3] and 355.0 kg [298.5–398.8] (p<0.000) and withers' height 132.5 cm [129.8–136.0] and 126.5 cm [121.0–129.5] (p<0.05) in S-P and P-P mares, respectively).

#### Body Condition Score (BCS)

BCS remained constant throughout gestation as illustrated in Figure 2B, with marked differences between obese pony mares (5.0 points [4.5–5.0]) and saddlebred and draft mares just above the superior limit for optimal body condition (3.75 points [3.25–4.0] and 3.75 points [3.5–4.0], respectively). Pony and saddlebred mares lost 0.5 and 0.4 point body score between the 11^th^ month of gestation and day 1 *postpartum*, whereas body scores remained unchanged in draft mares. BCS continued to fall during lactation, the loss being higher in pony mares (−1.5 point) compared to saddlebred and draft mares (−0.75 and −0.5 points). At weaning on day 180 *postpartum*, pony and saddlebred mares were at the lower limit for what is considered the optimal body condition (2.5 points [2.5–3.6] and 2.5 points [2.5–3.3]) whereas the draft mares' BCS had not really changed since the 5^th^ gestational month (3.25 points [3.0–3.8]).

#### Non Esterified Fatty Acids (NEFA)

Plasma NEFA concentrations reflect lipomobilization and increase in the case of negative energy balance. NEFA concentrations remained stable throughout gestation as illustrated in Figure 2C. There was no significant difference for NEFA between pony, saddlebred and draft mares on the 5^th^ and 9^th^ gestational months but NEFA concentrations were significantly higher in pony *vs* saddlebred (p<0.05) and draft mares (p<0.000). NEFA concentrations started to rise on the 9^th^ and 11^th^ month of gestation in pony mares and in saddlebred/draft mares, respectively. They reached their maximum 1 day after foaling in the 3 breeds. NEFA concentrations remained relatively stable throughout lactation, levels being significantly higher in pony mares at 5 months *postpartum* than in saddlebred mares at 6 months *postpartum* (p<0.05).

Pony and draft mares carrying saddlebred pregnancies tended to have higher NEFA concentrations at 5 months of gestation compared to pony and draft mares carrying pony pregnancies, respectively (p = 0.058 and p = 0.057). In contrast, after birth, pony mares suckling saddlebred foals had significantly lower plasma concentrations than those suckling control foals 5 months *postpartum* (p<0.05) but not before.

#### Leptin

Plasma leptin concentrations during pregnancy are illustrated in Figure 2D. Depending on times, they were significantly (p<0.000) or tended to be higher in pony *vs* saddlebred. They were significantly higher in pony *vs* draft mares (p<0.000) at all time points until parturition. Leptin remained stable after parturition, was low and not significantly different between breeds. Leptin concentrations fell from the 11^th^ month of gestation in all mares except for the 3 groups of mares carrying saddlebred pregnancies where they started to decrease at 5 (S-P) or 9 (S-D, S-S) months of gestation. They reached their lowest concentration on day 1 *postpartum* except for control pony mares where lowest plasma concentrations were reached on day 90 *postpartum*.

### Breed effects in control foals (P-P vs S-S)

**Figure 3:**
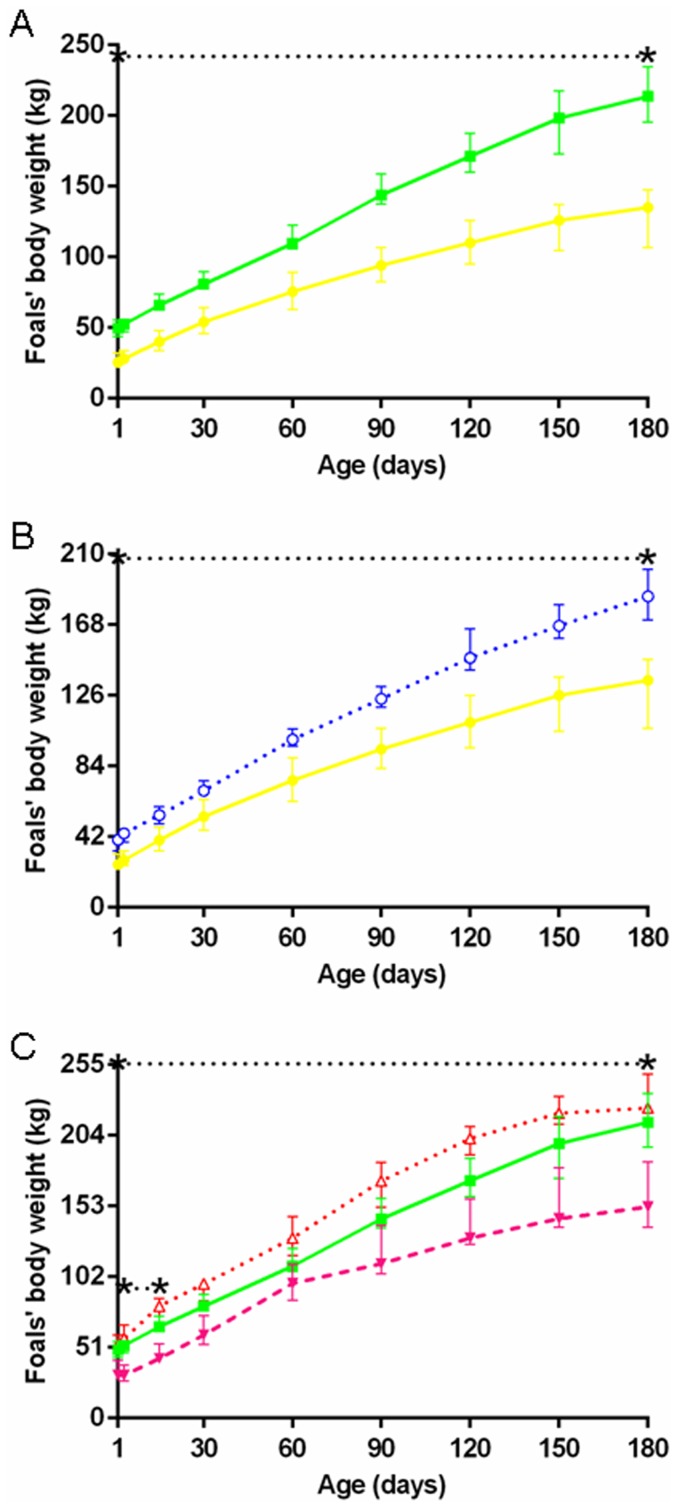
Foals' body weights from birth to weaning in the five groups. **A:** P-P (•) *vs* S-S (▪). **B:** P-P (•) *vs* P-D (○). **C:** S-P (▾) *vs* S-S (▪) *vs* S-D (Δ) (P-P: Pony in Pony, P-D: Pony in Draft, S-P: Saddlebred in Pony, S-S: Saddlebred in Saddlebred, S-D: Saddlebred in Draft). Curves are presented as medians and interquartile ranges. The median values between the asterisks differ significantly from each other (F1-LD-F1 model followed by Mann-Whitney or Kruskal-Wallis test, p<0.05). In graph C, median values under the lower and upper dotted lines between asterisks significantly differ between S-P and S-S and between S-P and S-D, respectively. NB: Different scales were used for A, B and C in order to show the differences.

P-P and S-S pregnancies lasted 331.9 days [326.7–337.7], with no significant breed effect. At birth, P-P foals were significantly lighter than S-S foals (25.5 kg [22.5–32.0] *vs* 49.4 kg [43.9–55.4], p<0.000). These body weight differences were maintained until day 180 and confirmed at each time point (Figure 3A).

**Figure 4:**
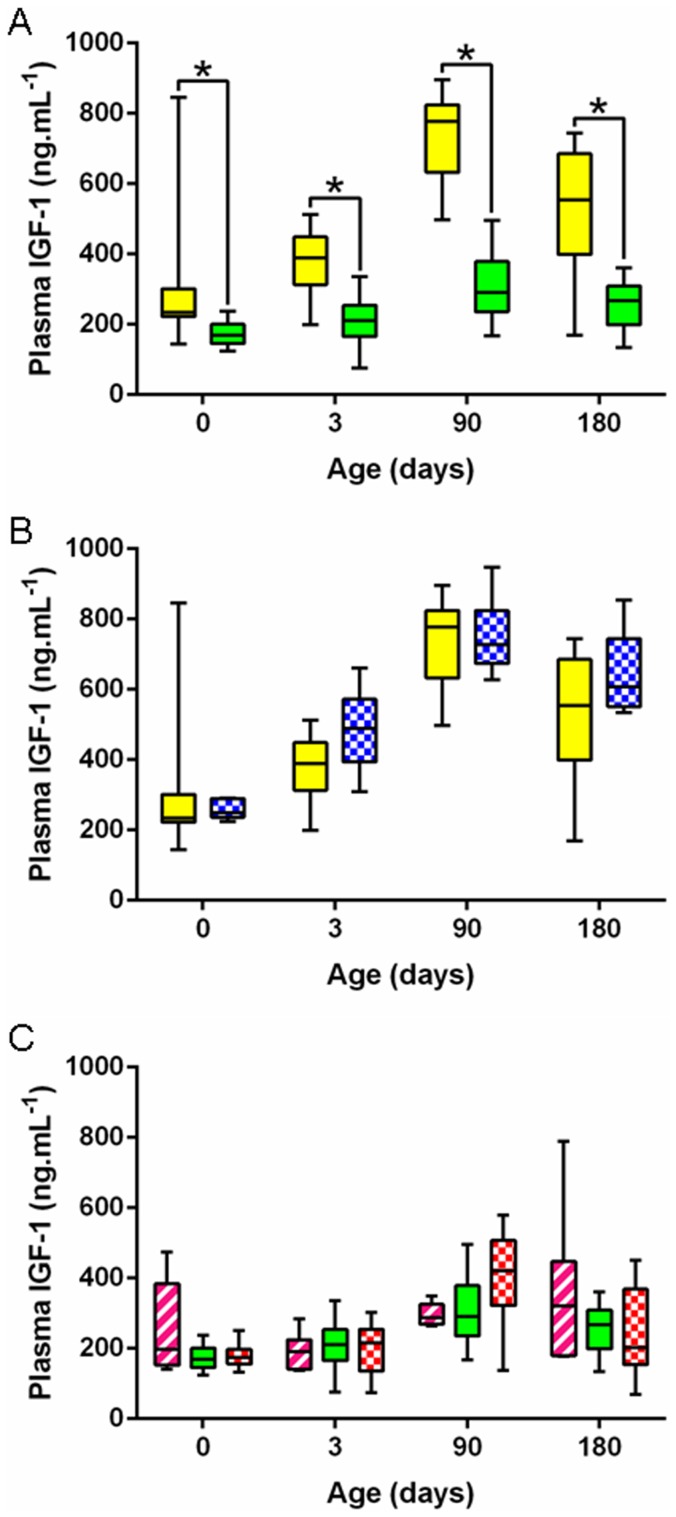
Foals' plasma IGF-1 levels from birth to weaning in the five groups. **A:** P-P (full yellow) *vs* S-S (full green). **B:** P-P (full yellow) *vs* P-D (chequered blue). **C:** S-P (striped pink) *vs* S-S (full green) *vs* S-D (chequered red) (P-P: Pony in Pony, P-D: Pony in Draft, S-P: Saddlebred in Pony, S-S: Saddlebred in Saddlebred, S-D: Saddlebred in Draft). Curves are presented as medians and interquartile ranges. The median values under the asterisks differ significantly from each other (F1-LD-F1 model followed by Mann-Whitney or Kruskal-Wallis test, p<0.05).

**Figure 5:**
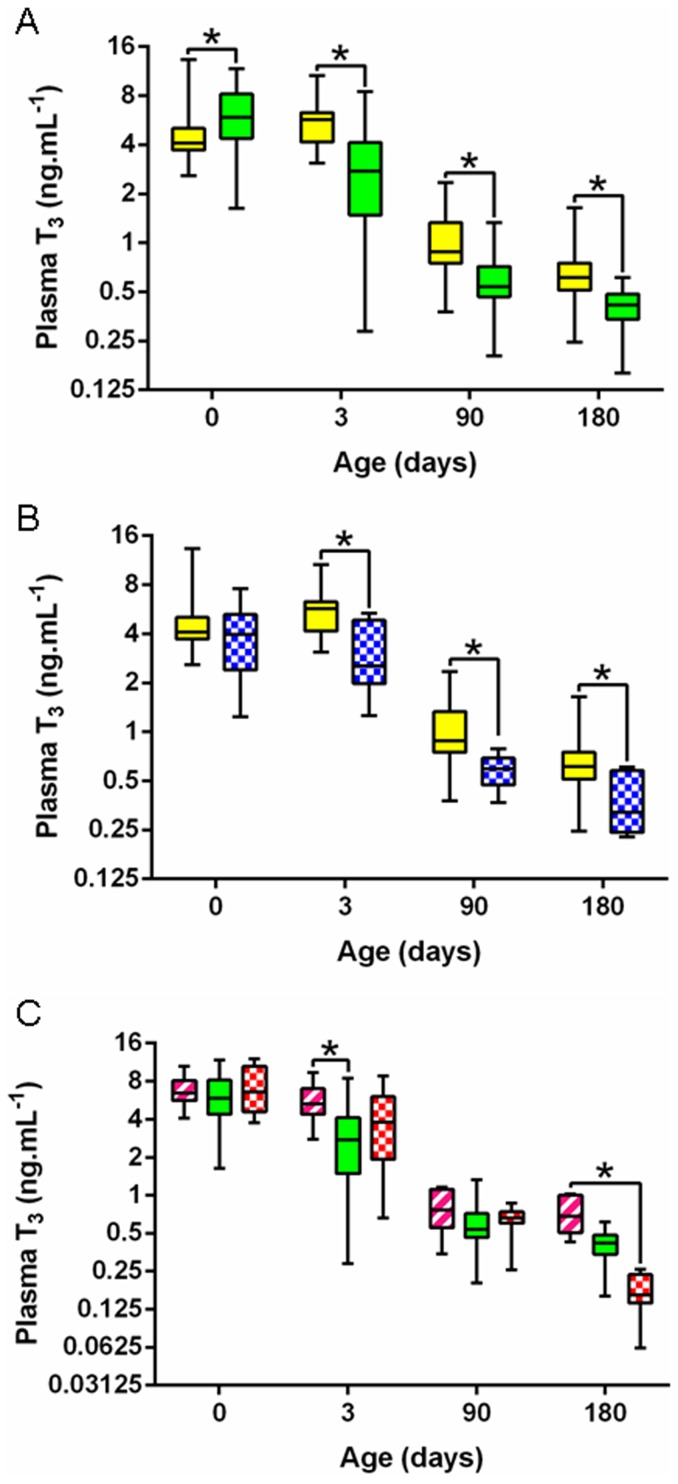
Foals' plasma T_3_ levels from birth to weaning in the five groups. **A:** P-P (full yellow) *vs* S-S (full green). **B:** P-P (full yellow) *vs* P-D (chequered blue). **C:** S-P (striped pink) *vs* S-S (full green) *vs* S-D (chequered red) (P-P: Pony in Pony, P-D: Pony in Draft, S-P: Saddlebred in Pony, S-S: Saddlebred in Saddlebred, S-D: Saddlebred in Draft). Curves are presented as medians and interquartile ranges and the scale on the y-axis is semi-logarithmic. The median values under the asterisks differ significantly from each other (F1-LD-F1 model followed by Mann-Whitney or Kruskal-Wallis test, p<0.05.

**Figure 6:**
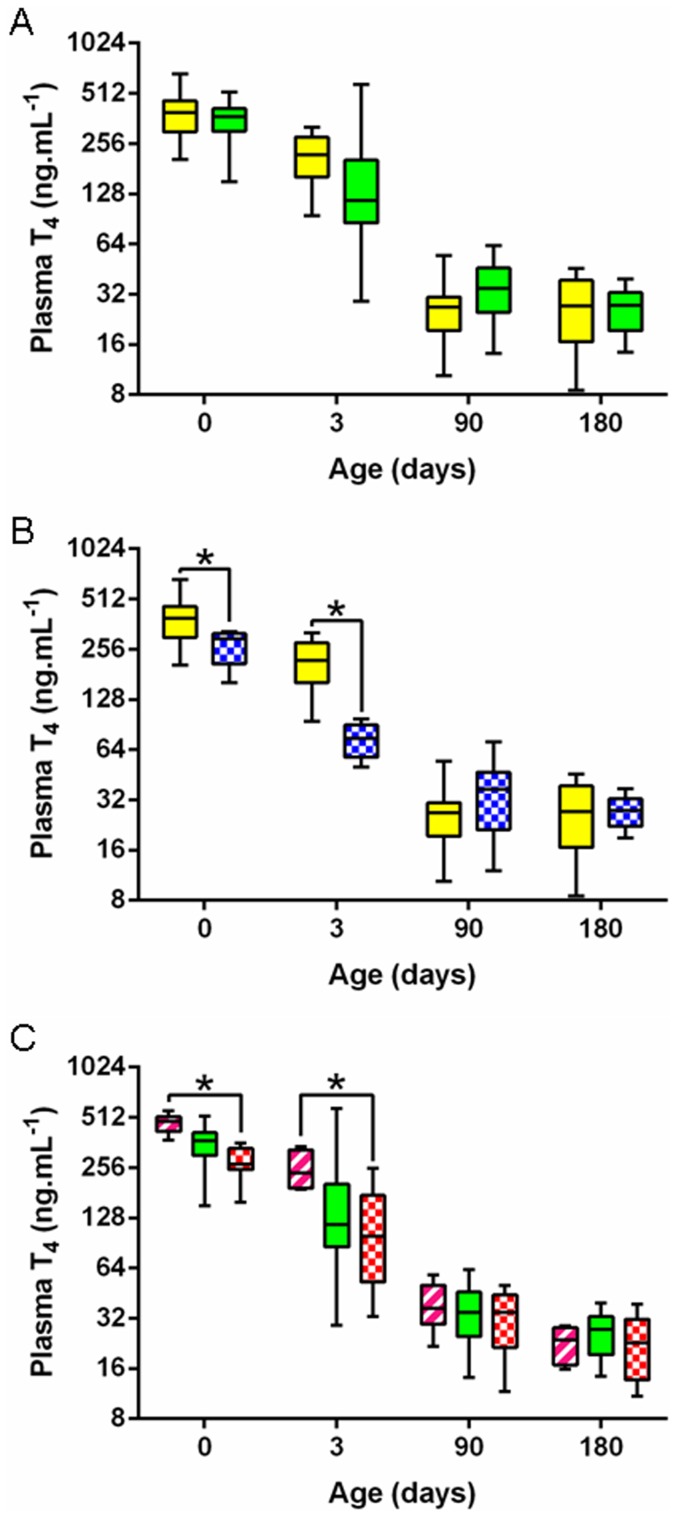
Foals' plasma T_4_ levels from birth to weaning in the five groups. **A:** P-P (full yellow) *vs* S-S (full green). **B:** P-P (full yellow) *vs* P-D (chequered blue). **C:** S-P (striped pink) *vs* S-S (full green) *vs* S-D (chequered red) (P-P: Pony in Pony, P-D: Pony in Draft, S-P: Saddlebred in Pony, S-S: Saddlebred in Saddlebred, S-D: Saddlebred in Draft). Curves are presented as medians and interquartile ranges and the scale on the y-axis is semi-logarithmic. The median values under the asterisks differ significantly from each other (F1-LD-F1 model followed by Mann-Whitney or Kruskal-Wallis test, p<0.05).

IGF-1 concentrations were strongly related to the foal's breed and were significantly higher in P-P *vs* S-S foals at all time points from birth to day 180 (significant group effect, p<0.000) (Figure 4A). A significant group effect was also observed for T_3_ concentrations (p<0.000). T_3_ concentrations were significantly lower at birth (Figure 5A) (p<0.005) but were significantly higher on days 3, 90 and 180 (p<0.000) in P-P *vs* SS foals. No significant group effect was observed for T_4_ concentrations (Figure 6A) and, as a result, T_3_/T_4_ ratio was significantly different between breeds (p<0.000). T_3_/T_4_ ratio was significantly decreased at birth (p<0.05), unaffected on day 3 and significantly increased on days 90 and 180 in P-P *vs* S-S foals (p<0.000 and p<0.005).

**Figure 7:**
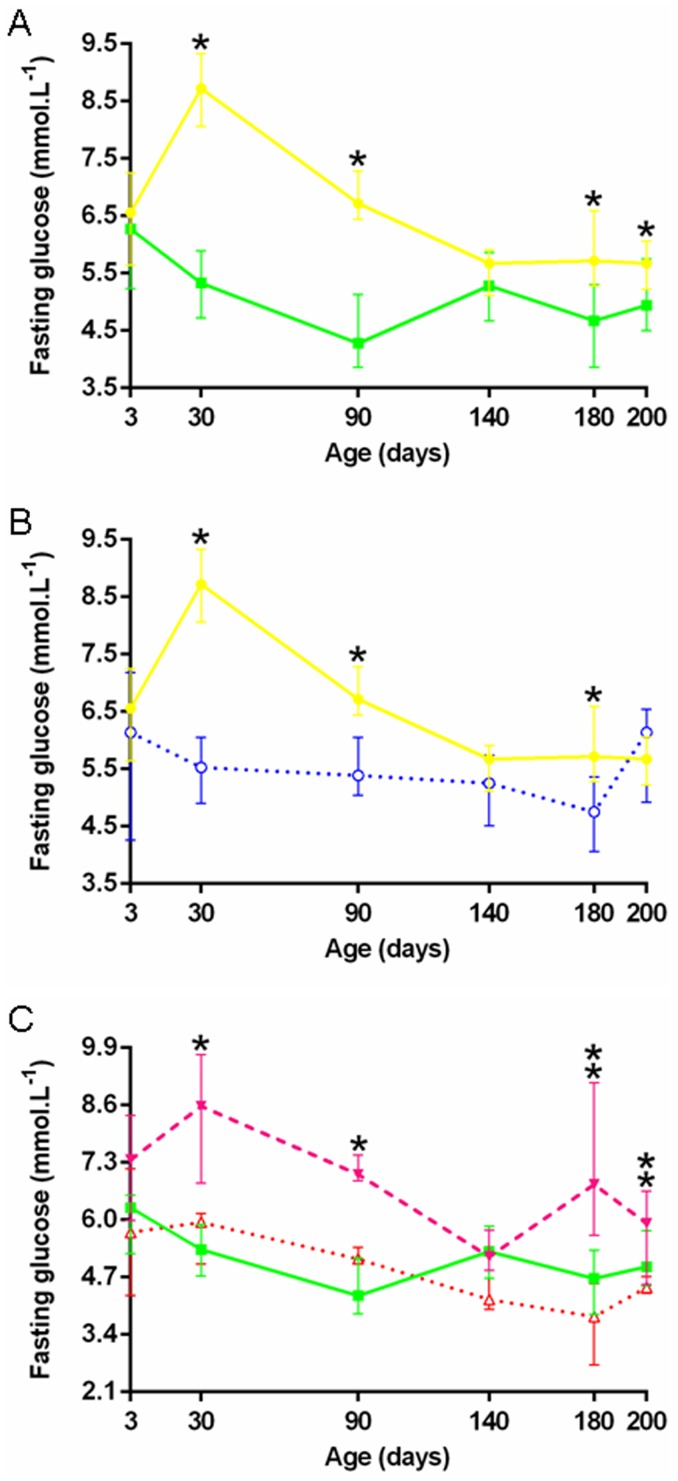
Foals' fasting glucose from birth to weaning in the five groups. **A:** P-P (•) *vs* S-S (▪). **B:** P-P (•) *vs* P-D (○). **C:** S-P (▾) *vs* S-S (▪) *vs* S-D (Δ) (P-P: Pony in Pony, P-D: Pony in Draft, S-P: Saddlebred in Pony, S-S: Saddlebred in Saddlebred, S-D: Saddlebred in Draft). Curves are presented as medians and interquartile ranges. The median values under the asterisks differ significantly from each other (F1-LD-F1 model followed by Mann-Whitney or Kruskal-Wallis test, p<0.05). In graph C, median values under the simple and double asterisks significantly differ between S-P and S-S and between S-P and S-D, respectively.

**Figure 8:**
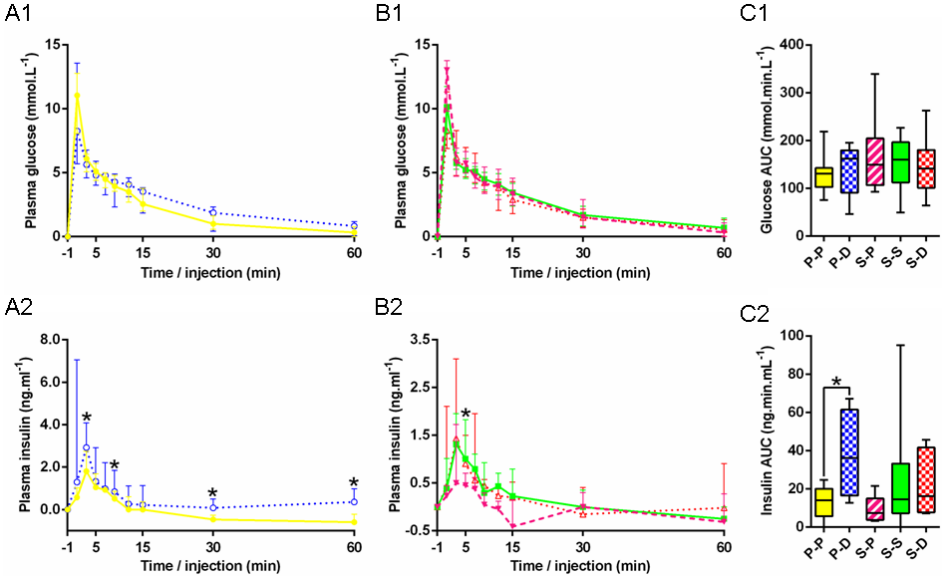
Changes in the plasma concentrations of glucose and insulin in response to glucose bolus in the five groups. **A:** Glycemia (A1) and insulinemia (A2) in P-P (•) *vs* P-D (○). **B:** Glycemia (B1) and insulinemia (B2) in S-P (▾) *vs* S-S (▪) *vs* S-D (Δ). **C:** Area under the curve for glucose (C1) and insulin (C2) in P-P (full yellow), P-D (chequered blue), S-P (striped pink), S-S (full green) and S-D (chequered red) (P-P: Pony in Pony, P-D: Pony in Draft, S-P: Saddlebred in Pony, S-S: Saddlebred in Saddlebred, S-D: Saddlebred in Draft). Curves are presented as medians and interquartile ranges. The median values under the asterisks differ significantly from each other (F1-LD-F1 model followed by Mann-Whitney or Kruskal-Wallis test, p<0.05). In graph B2, the asterisk indicates a significant difference between S-P and S-S.

Fasting glucose was also breed specific with a significant group effect (p<0.000). P-P foals had significantly higher fasting glucose than S-S foals, except on days 3 and 140 (Figure 7A). During IVGTT on day 3, plasma glucose AUC tended to be lower in P-P *vs* S-S foals (131.0 mmol/min/L [102.8–142.8] *vs* 160.0 mmol/min/L [112.3–196.6], p = 0.056). Other parameters remained mainly unaffected (Figure 8). Clamps on day 200, however, highlighted breed specific glucose metabolism rates (M): glucose metabolism was significantly reduced in P-P *vs* S-S foals (0.013 mmol/kg/min [0.011–0.018] *vs* 0.020 mmol/kg/min [0.014–0.030], p<0.001, Table S3), indicating increased insulin resistance in pony *vs* saddlebred foals.

### Sexual dimorphism in control foals

Data were analyzed for sexual dimorphism in 12 female *vs* 9 male control pony foals and in 16 *vs* 12 male control saddlebred foals. In both breeds, fillies and colts had similar gestational length and weight until 6 months of age. Saddlebred fillies had significantly higher IGF-1 concentrations on day 90 (p<0.05) and higher T_3_ concentrations on days 3 and 90 (p = 0<0.05 for both) than saddlebred colts. In pony foals, significantly decreased T_4_ levels were observed in pony fillies *vs* colts on day 180 (p<0.05). There was a significant effect of the sex on fasting glucose in saddlebreds with increased concentrations in fillies *vs* colts on days 90 and 140 (p<0.01). In contrast, fasting glucose in pony foals remained unaffected by the sex. No significant sex effect was found for IVGTT and clamps in either of the two breeds. Because the sex ratio was unbalanced, sex specificities were not investigated within experimental groups.

### Effect of increased fetal growth in pony foals

Although not significant when both breeding seasons were analyzed together, it should be noted that, in the first breeding season, P-D pregnancies (332.1 days [321.7–333.1]) were significantly shorter compared to P-P pregnancies (339.1 days [334.3–343.1], p<0.05). Altogether, P-D foals (40.1 [33.6–40.9] kg) had a significantly 57.3% increased birth weight (p<0.000) compared to P-P controls and remained significantly heavier until day 180 where they still had a significantly increased body weight (+37.0%, p<0.000) (Figure 3B).

IGF-1 concentrations remained unaffected by transfer into a draft mare except on day 3 where P-D foals had significantly higher plasma concentrations than P-P controls (p<0.05) (Figure 4B). T_3_ concentrations were significantly reduced in P-D *vs* P-P foals on days 3, 90 and 180 (p<0.000) (Figure 5B) whereas T_4_ concentrations were significantly reduced only on days 0 and 3 (p<0.05 and p<0.000, respectively) (Figure 6B). T_3_/T_4_ ratios were subsequently significantly increased on day 3 (p<0.05) and decreased on days 90 and 180 (p<0,05) in P-D *vs* P-P foals.

Fasting glucose was affected the same way, with significantly reduced plasma concentrations on days 30, 90 and 180 (p<0.005) in P-D *vs* P-P foals (Figure 7B). No significant group effect was found on glucose parameters during IVGTT on day 3 (Figure 8) nor during clamps on day 200 (Table S3). P-D foals, however, had significantly higher insulin AUC (p<0.05, Figure 8C2), higher plasma insulin increments at 3, 9, 30 and 60 minutes (p<0.05, Figure 8A2), as well as higher maximal insulin increments (p<0.05) compared to P-P foals.

### Effect of increased or reduced fetal growth in saddlebred foals

S-P pregnancies were significantly longer compared to S-S (344.0 days [334.5–353.8] *vs* 330.8 days [325.9–336.3], respectively, p = 0.05) and S-D pregnancies (328.0 days [327.0–334.1], p<0.05) pregnancies. There was no significant difference in gestational length in S-D *vs* S-S.

Body weight in S-P, S-S and S-D foals are represented in Figure 3C. S-P foals (31.0 kg [28.0–41.5]) tended to be lighter at birth compared to S-S controls (−37.2%, p = 0.078). They remained significantly lighter than S-S controls until day 30 (p<0.000) at which time the difference was no longer significant, although S-P bodyweight at 6 months of age was significantly less by 29% compared to S-S controls. S-P foals were also significantly lighter than S-D foals from birth to 180 days (p<0.000), with a significantly lighter birth weight (−42.3%, p<0.000). In contrast, the bodyweights of S-D foals were not significantly different compared to S-S controls.

IGF-1 concentrations in saddlebred foals were not affected by transfer into either a pony or a draft mare (Figure 4C). S-P foals only differed from S-S controls by elevated T_3_ concentrations on day 3 (p<0.05, Figure 5C). There was no difference between S-P and S-S foals for T_4_ (Figure 6C) and T_3_/T_4_ ratio. In contrast, T_3_ concentrations were significantly increased on day 180 (p<0.05) and T_4_ concentrations were significantly increased on days 0 and 3 (p<0.000 and p<0.05) in S-P *vs* S-D foals (Figures 5C and 6C), resulting in significantly higher T_3_/T_4_ ratios on day 180 in S-P *vs* S-D foals (p<0.000). Saddlebred foals were not affected by transfer into a draft mare with no significant difference between S-D and S-S foals.

Fasting plasma glucose was significantly higher in S-P *vs* S-S foals on days 30 and 90 (p<0.005) and in S-P *vs* S-D foals on days 180 and 200 (p<0.000 and p<0.05) (Figure 7C). No significant group effect was found for plasma glucose AUC during IVGTT on day 3 but the maximal increment in glucose was significantly higher in S-P *vs* S-D foals (13.0 mmol/L [9.8–13.8] *vs* 8.3 mmol/L [6.9–11.3], p<0.05, Figure 8B1). The maximum insulin increment tended to be reduced in S-P compared to S-D foals (p = 0.081). Clamps demonstrated no difference in S-P *vs* S-S or in S-D *vs* S-S foals. But S-P and S-D differed from each other by increased M in S-P on day 200 (0.025 mmol/kg/min [0.020–0.035] *vs* 0.016 mmol/kg/min [0.013–0.020], p<0.05, Table S3).

## Discussion

In the present study, we have confirmed that ponies can not be considered miniature versions of saddlebreds. Ponies were systematically fatter than saddlebreds, as confirmed by higher BCS and plasma leptin concentrations in pony mares, inducing confounding factors during pregnancy between obese pony and normal weight saddlebred and draft mares. In foals, significantly higher plasma IGF-1 and T_3_ concentrations were observed in ponies *vs* saddlebreds in the first six months of age. Ponies also appeared to have higher fasting glycemia at most times and reduced glucose metabolism at 6 months compared to saddlebreds. Little sexual dimorphism was observed in both breeds on the parameters studied here.

Reduced fetal growth induced by transfer of saddlebred embryos into pony mares resulted in reduced weight until one month of age. IGF-1 concentrations remained unchanged by embryo transfer. T_3_ concentrations were increased shortly after birth compared with saddlebred controls. Moreover, “restricted” S-P foals had higher fasting glucose concentrations. Direct comparison with “enhanced” S-D foals highlighted that S-P foals had increased fasting glucose but a tendency towards reduced insulin secretion with unaffected glucose clearance after IVGTT, indicating increased glucose tolerance and increased insulin sensitivity, respectively, as well as a higher glucose metabolism at 6 months of age, confirming increased insulin sensitivity.

Enhanced fetal growth affected the ponies more than the saddlebreds, possibly due to a larger difference in body size between ponies and draft mares compared to saddlebreds and draft mares. P-D foals remained heavier than their pony controls until weaning and had significantly reduced T_3_ and T_4_ concentrations. IGF-1 concentrations remained unchanged by embryo transfer. Fasting glucose was decreased at most times and early glucose tolerance tests indicated insulin resistance in “enhanced” neonatal foals compared to control ponies in which insulin resistance developed at 6 months of age.

One limitation of this study is that control groups were produced by artificial insemination, whereas experimental groups were produced by embryo transfer. Although data are lacking in the horse, it has been previously shown in humans and in rodent models that assisted reproductive technologies such as *in vitro* fertilization and/or ovarian hyperstimulation as well as culture media could lead to imprinting disorders and abnormalities in post-natal growth, body composition, glucose metabolism, behavior or systolic blood pressure in adult offspring [34]–[38]. Combined effects of hyperstimulation and embryo treatment have been demonstrated on *H19* gene imprinting [35], [36] but it is still unclear whether embryo transfer as such with limited embryo culture time induces long term effects. In the present study, hyperstimulation was not used. Embryos were maintained in culture media in an Equitainer for a maximum of 6 hours before transfer as usually performed in practice.

Elliott et al (2009) showed that parity was the main factor affecting birthweight, with a limited impact of age in Thoroughbred horses [39]. Here, draft mares were significantly younger than the two other breeds which could result in foals with a reduced birth weight. This did not prevent P-D foals from being significantly heavier than P-P. On the other hand, saddlebred mares had a significantly higher parity compared to both other groups, which may have caused the increased birth weight of S-S foals. The combined effects of young draft mares and higher parity of saddlebred mares may have contributed to the lack of effect in S-D foals. Due to the low number of animals in some groups, it was not possible to test this hypothesis with statistical analysis. However, correction of the data with regards to parity according to Elliott et al (2009) (+0.7 kg per each unit increase in parity) did not alter the results on foal weight at birth [39].

The metabolism of pony mares was different from that of the other mares used in this study: pony mares could be considered as obese in the beginning of the project, with maximal BCS of 4.5 to 5. Indeed, ponies have in general higher BCS, are more resistant to insulin than standardbred horses [29], [40], [41], possess higher plasma insulin and leptin concentrations [22] and express components of the equine metabolic syndrome [42]. These metabolic characteristics are the source of confounding factors in this study where the smaller breed was also metabolically different. Unfortunately, fasting blood samples were not collected from the mares before pregnancy so it is not possible to confirm hyperinsulinemia in non-pregnant pony mares, although excess BCS is associated with decreased insulin sensitivity in horses [43], [44]. Plasma leptin concentrations were similar to that reported by others in pregnant mares [30], [45]–[47]. Although seasonal variations have been observed in horses [48], all mares were collected at the same time in the season over the two years and also at the same time in the day (morning), thus reducing the variability due to the environment. Leptin concentrations started to decrease earlier in gestation in S-P mares, indicating that the burden of carrying a large fetus may have induced earlier lipomobilization in pony mares, although NEFA only increased at 9 months of pregnancy and BCS remained stable until just prior to foaling. As shown by others, maternal plasma leptin decreased sharply after birth in all groups, together with increased NEFA and a progressive reduction in BCS associated with lactation [49]. The rapid *postpartum* decrease in circulating leptin may be due to a loss of placental leptin because placental leptin mRNA expression has been reported in humans [50], [51], rats [52] and sheep [53]. Unpublished data from our laboratory however indicates that the equine placenta does not express leptin. A *postpartum* reduction in circulating leptin concentrations has been reported in humans [50], [51] and Japanese monkeys [54], but not in rats [52] or sheep [55].

In the present study, birth weights were significantly increased by 57% in enhanced P-D foals and decreased by 37% in restricted S-P foals. This is consistent with enhanced Pony-in-Thoroughbred and restricted Thoroughbred-in-Pony foals where a 15% increase and reduction in body dimensions, respectively, were reported [56]. Growth profiles from both enhanced and restricted foals differed from their respective breed controls, with P-D remaining heavier than P-P and S-P remaining lighter than S-D foals, in contrast to what was reported in the pony and thoroughbred embryo transfer experiments where differences had disappeared by 6 months of age [56]. The effects on weight gain were probably higher in the present study because of the bigger size difference between the breeds. Although catch-up growth is often observed in IUGR animals [57]–[60], this was not observed in this study, probably due to the limited milk production in pony mares. Similarly, increased milk production in draft mares could account for the growth advantage in P-D foals, since milk yields are known to be breed specific [61] and to be increased with the mare's size [61]. Hormones and growth factors such as T_3_ [62], leptin [63], IGF-1 and insulin [64] and thyroid stimulating hormone (TSH) [49] are also supplied through the mare's colostrum. In Quarter horses, milk leptin, IGF-1 and TSH concentrations were at their maximum the day of parturition and reached minimum at 2 months *postpartum* (leptin and TSH) or became undetectable by 12 days *postpartum* (IGF-1) [49]. Those elements moderate the importance of the genetic growth potential, highlighting the importance of the effects of the pre- and post-natal environments on growth until weaning.

Glucose homeostasis depends on both the secretion of insulin by the pancreatic β cells and the sensitivity of skeletal muscles and adipose tissue to insulin. Although a slight sexual dimorphism was observed in saddlebred foals for fasting glucose (with fillies having a slightly more elevated fasting glycemia compared to colts), no other difference related to sex was observed, maybe due to the reduced number of animals in this study. Here, restricted foals were growth retarded compared to their own breed counterparts and appeared slightly dysmature although their gestation length was increased. Dysmaturity, which shares many clinical characteristics with prematurity [33], is associated with a reduced insulin secretion in the immediate post-natal period compared to full term foals [65]. Indeed, insulin secretion tended to be reduced in S-P foals at 3 days of age but fasting glucose was increased at most times, suggesting insulin dysregulation. As also described in one month old sheep [66], [67], glucose metabolism was increased in S-P foals at 6 months of age, indicating increased insulin sensitivity, which is in agreement with data in several species showing that IUGR in the absence of post-natal catch-up growth improves insulin sensitivity [57], [68], [69]. In horses, pancreatic maturation is complete around 3 months of age [70], so changes observed at 6 months should not be associated with pancreatic immaturity. In contrast, as also shown previously in ponies transferred into thoroughbred recipient mares [27], P-D had increased β cells response to a glucose bolus compared to P-P foals. Subsequently, P-D had lower fasting plasma glucose concentrations than P-P until 6 months of age although insulin sensitivity remained normal as demonstrated by clamps. S-D foals followed a similar trend for glucose metabolism as observed with P-D foals. Differences were not as marked when compared with their normal size S-S controls but were mostly significant when they were compared with the IUGR S-P. This suggests that these effects were not related to the breed but mainly to the experimental manipulation of growth.

IGF-1 and thyroid hormones are some of the major hormonal factors involved in post-natal growth. IGF-1 is one of the most important regulators of growth in the newborn, mediating most effects of growth hormone (GH). Plasma IGF-1 concentrations, although strongly related to the foal's breed and higher in pony compared to saddlebred foals, were consistent with previously published data [30], [49], [71] and followed similar trends, with increased concentrations between birth and 3 months of age, as described elsewhere [64], [71], [72]. In humans, IUGR babies have low plasma concentrations of IGF-1 [73]. In horses, bottled fed foals have lower plasma IGF-1 concentrations compared to those nursing on the mare [71], [72], but Panzani *et al.* found no statistical differences in plasma IGF-1 concentrations between sick, induced or naturally delivered foals [72]. Neither reduction nor enhancement of *prepartum* growth affected IGF-1 in the present study.

Thyroid hormones play a crucial role in energy metabolism, thermoregulation, metabolism of nutriments and inorganic ions and for stimulation of growth. They optimize the action of catecholamines and stimulate the synthesis and action of IGF-1 and GH [74]. Plasma T_3_ concentrations were breed-related, being higher in pony *vs* saddlebred foals, whereas plasma T_4_ concentrations remained unchanged between breeds. This is consistent with previous work demonstrating that plasma T_3_ and T_4_ concentrations differ between breeds of horses, with no correlation with adult body size and no obvious correlation with physiological status [75]. Thyroid hormones concentrations at birth in foals are higher than at any physiological age in any species and it has been hypothesized that this could be due to the high thermogenic capacity and the rapid growth in this species [76]. Here, growth-enhanced P-D foals had decreased T_4_ concentrations in the immediate *postpartum* period and decreased T_3_ concentrations from birth to weaning compared to P-P controls. Interestingly, increased weight gain is observed in hypothyroid patients [77], as was observed in these foals. In contrast, S-P foals had elevated T_4_ and T_3_ concentrations in the first days following parturition compared to saddlebred controls. Since increases in circulating T_3_ in the immediate post-natal period were shown to be closely related to adrenocortical activity [78], an increased stress *in utero* in S-P foals due to IUGR may have contributed to the increased neonatal T_3_ concentrations in this group. In older individuals, hyperthyroidism is accompanied by an increased metabolic rate, increased thermogenesis and weight loss despite increased food intake [77], [79], [80]. The increased metabolic rate observed through the clamps in the S-P foals is in agreement with the increased thyroid hormones. Moreover, since about 80% of T_3_ is produced by the hepatic deiodination of T_4_ [81], the increased T_3_/T_4_ ratio observed in S-P foals probably reflects increased hepatic deiodinase activity and the contrary is observed in P-D foals.

In conclusion, this work demonstrates that the modification of fetal growth through the transfer of large/small breed embryos into recipients of a small/large breed modifies post-natal growth and thyroid hormones profiles with no catch-up growth at least until weaning. Moreover, glucose metabolism is affected, which may affect further capacity to perform in equestrian sports. Although long term effects have not been studied here, data obtained in other species and in humans strongly indicate that fetal IUGR and fetal overgrowth both induce increased susceptibility to metabolic diseases in adulthood [82]. This may be of importance in the presence of an increasing prevalence of the equine metabolic syndrome [42], [83].

## Supporting Information

Table S1 **Nutritional value of the diets on farms 1 and 2.**
(DOC)

Table S2 **Mares' parameters measured in the five groups.**
(DOC)

Table S3 **Foals' parameters measured in the five groups.**
(DOC)
