# Supplementary material for: Enhanced or Reduced Fetal Growth Induced by Embryo Transfer into Smaller or Larger Breeds Alters Post-Natal Growth and Metabolism in Pre-Weaning Horses
Source: PLoS One. 2014 Jul 9;9(7):e102044. doi: 10.1371/journal.pone.0102044 (PMC4090198; doi:10.1371/journal.pone.0102044)
Supplement: Table S1 — Nutritional value of the diets on farms 1 and 2. (DOC) [file pone.0102044.s001.doc]

| **Table S1. Nutritional value of the diets on farms 1 and 2.** | | | | | |
| --- | --- | --- | --- | --- | --- |
| **FARM 1 IN NOUZILLY** | | | | | |
|  | | | | | |
| **PREGNANT AND LACTATING PONY MARES** | **DM (kg)** | **UFC** | **MADC (g)** | **DM/UFC** | **MADC/UFC** |
| **Ovulation to gestational month 5** | Pasture | | | | |
| **Gestational months 5 to 8** | 5,23 | 2,3 | 158,51 | 2,28 | 69,16 |
| **Gestational month 9** | 5,30 | 2,9 | 208,00 | 1,83 | 71,72 |
| **Gestational month 10** | 4,50 | 2,8 | 169,22 | 1,58 | 59,48 |
| **Gestational month 11** | 4,50 | 2,8 | 169,00 | 1,61 | 60,36 |
| **First 10 days after foaling (P-P mares in 2011 and 2012 and S-P mares in 2012)** | 10,11 | 6,4 | 459,03 | 1,59 | 72,01 |
| **First 2 months after foaling (S-P mares in 2011)** | 9,00 | 5,4 | 489,00 | 1,66 | 90,01 |
| **Until weaning at age 6 months** | Pasture | | | | |
|  |  |  |  |  |  |
| **POST-WEANING P-P and S-P FOALS** | **DM (kg)** | **UFC** | **MADC (g)** | **DM/UFC** | **MADC/UFC** |
| **Age 7 to 12 months (P-P foals born in 2011 and 2012)** | 3,90 | 2,8 | 228,00 | 1,41 | 81,99 |
| **Age 6 months (S-P foals born in 2011)** | 4,90 | 2,5 | 129,61 | 1,99 | 52,76 |
| **Age 7 months (S-P foals born in 2011)** | 4,90 | 3,3 | 358,00 | 1,50 | 109,25 |
| **Age 7 to 8 months (S-P foals born in 2012)** | 5,30 | 4,4 | 446,91 | 1,21 | 101,77 |
|  | | | | | |
|  | | | | | |
|  | | | | | |
|  | | | | | |
|  | | | | | |
|  | | | | | |
|  | | | | | |
|  | | | | | |
|  | | | | | |
|  | | | | | |
|  | | | | | |
|  | | | | | |
|  | | | | | |
|  | | | | | |
|  | | | | | |
|  | | | | | |
|  | | | | | |
|  | | | | | |
|  | | | | | |
|  | | | | | |
|  | | | | | |
|  | | | | | |
|  | | | | | |
|  | | | | | |
| **FARM 2 IN CHAMBERET** | | | | | |
|  | | | | | |
| **PREGNANT AND LACTATING SADDLEBRED MARES** | **DM (kg)** | **UFC** | **MADC (g)** | **DM/UFC** | **MADC/UFC** |
| **Ovulation to gestational month 5** | Pasture | | | | |
| **Gestational month 5** | 8,29 | 5,9 | 344,5 | 1,42 | 58,89 |
| **Gestational month 6** | 9,70 | 7,3 | 448,0 | 1,33 | 61,37 |
| **Gestational month 7** | 9,80 | 8,3 | 497,0 | 1,18 | 59,88 |
| **Gestational month 8** | 9,10 | 7,3 | 463,6 | 1,24 | 63,23 |
| **Gestational months 9 and 10** | 9,50 | 7,1 | 601,0 | 1,34 | 84,65 |
| **Gestational month 11** | 9,1 | 6,7 | 540 | 1,36 | 80,60 |
| **First 3 days after foaling** | 15,4 | 12,5 | 1364,5 | 1,23 | 109,16 |
| **Until weaning at age 6 months** | Pasture | | | | |
|  | | | | | |
| **PREGNANT AND LACTATING DRAFT MARES** | **DM (kg)** | **UFC** | **MADC (g)** | **DM/UFC** | **MADC/UFC** |
| **Ovulation to gestational month 5** | Pasture | | | | |
| **Gestational month 5** | 7,60 | 5,8 | 440,00 | 1,31 | 75,86 |
| **Gestational month 6** | 9,00 | 7,1 | 446,50 | 1,28 | 63,33 |
| **Gestational month 7** | 9,80 | 8,3 | 497,00 | 1,18 | 59,88 |
| **Gestational month 8** | 9,60 | 7,3 | 456,00 | 1,32 | 62,47 |
| **Gestational months 9 and 10** | 8,60 | 8,4 | 732,00 | 1,02 | 87,14 |
| **Gestational month 11** | 10,6 | 8,5 | 622 | 1,25 | 73,14 |
| **First 3 days after foaling** | 14,7 | 10,9 | 976 | 1,35 | 89,54 |
| **Until weaning at age 6 months** | Pasture | | | | |
|  | | | | | |
| **POST-WEANING P-D FOALS** | **DM (kg)** | **UFC** | **MADC (g)** | **DM/UFC** | **MADC/UFC** |
| **Age 7 to 12 months (P-D born in 2011)** | 3,05 | 1,5 | 143,00 | 2,09 | 97,95 |
| **Age 7 and 8 months (P-D born in 2012)** | ? | 1,9 | 208,73 | ? | ? |
|  | | | | | |
| **POST-WEANINNG S-S AND S-D FOALS** | **DM (kg)** | **UFC** | **MADC (g)** | **DM/UFC** | **MADC/UFC** |
| **Age 6 to 12 months (S-S and S-D born in 2011)** | 3,05 | 1,5 | 143,00 | 2,09 | 97,95 |
| **Age 6 to 12 months (S-S born in 2012)** | 4,93 | 1,9 | 208,73 | 2,57 | 108,71 |

DM: dry matter, UFC: horse feed unit, MADC: horse digestible crude protein. P-P: Pony in Pony, P-D: Pony in Draft, S-P: Saddlebred in Pony, S-S: Saddlebred in Saddlebred, S-D: Saddlebred in Draft.
