# Supplementary material for: Enhanced or Reduced Fetal Growth Induced by Embryo Transfer into Smaller or Larger Breeds Alters Post-Natal Growth and Metabolism in Pre-Weaning Horses
Source: PLoS One. 2014 Jul 9;9(7):e102044. doi: 10.1371/journal.pone.0102044 (PMC4090198; doi:10.1371/journal.pone.0102044)
Supplement: Table S2 — Mares' parameters measured in the five groups. (DOC) [file pone.0102044.s002.doc]

**Table S2. Mares’ parameters measured in the five groups.**

|  | | P-P | P-D | S-P | S-S | S-D |
| --- | --- | --- | --- | --- | --- | --- |
| Gestation length (days) | | 334.0 [328.0-340.1] | 327.7 [321.5-332.7] | 344.0 [334.5-353.8] | 330.8 [325.9-336.3] | 328.0 [327.0-334.1] |
| Body weight (kg) | 5^th^ gestational month | 359.0 [298.5-402.0] | 816.0 [762.0-877.0] | 400.0 [373.5-420.8] | 622.6 [559.6-652.4] | 792.0 [749.3-854.8] |
|  | 11^th^ gestational month | 356.0 [299.0-395.5] | 840.0 [768.1-913.3] | 405.0 [380.8-433.5] | 646.0 [607.3-685.6] | 842.9 [783.0-882.4] |
|  | 1 day *postpartum* | 328.0 [277.5-366.5] | 772.2 [708.6-821.3] | 378.0 [322.8-390.3] | 578.3 [538.3-597.1] | 761.7 [712.4-780.9] |
|  | 6 months *postpartum* | 325.0 [263.0-354.5] | 761.6 [735.2-825.9] | 361.5 [333.8-375.8] | 550.5 [531.4-597.8] | 697.3 [667.1-722.4] |
| Body score | 5^th^ gestational month | 5.00 [4.75-5.00] | 3.88 [3.56-4.06] | 5.00 [4.25-5.00] | 3.75 [3.25-3.88] | 3.88 [3.13-4.00] |
|  | 11^th^ gestational month | 4.50 [4.00-5.00] | 3.75 [3.19-4.00] | 4.75 [4.13-5.00] | 3.63 [3.19-3.75] | 3.75 [3.19-4.13] |
|  | 1 day *postpartum* | 4.00 [3.75-4.75] | 3.63 [3.00-3.81] | 4.00 [3.75-4.50] | 3.25 [3.00-3.75] | 3.75 [3.50-4.50] |
|  | 6 months *postpartum* | 3.00 [2.50-4.25] | 3.38 [3.00-4.06] | 2.50 [2.00-2.50] | 2.50 [2.38-3.38] | 3.00 [2.25-3.56] |
| Plasma non esterified fatty acids (µmol.L-1) | 5^th^ gestational month | 127.0 [90.0-182.5] | 110.0 [66.0-155.0] | 248.5 [119.5-402.3] | 136.0 [109.8-307.0] | 165.5 [135.3-194.3] |
|  | 11^th^ gestational month | 359.0 [175.0-819.5] | 124.5 [106.5-177.3] | 344.0 [190.8-563.5] | 112.0 [102.0-181.5] | 151.5 [108.5-199.5] |
|  | 1 day *postpartum* | 272.0 [155.0-548.3] | 611.0 [341.0-724.0] | 525.5 [224.0-827.0] | 340.5 [239.0-489.3] | 451.5 [218.8-876.5] |
|  | 5 or 6 months *postpartum* | 523.0 [446.0-637.3] | 497.0 [408.5-576.5] | 354.5 [325.0-384.0] | 254.0 [141.5-453.3] | 670.0 [33.00-708.0] |
| Plasma leptin (ng.mL^-1^) | 5^th^ gestational month | 6.65 [5.75-8.35] | 3.57 [2.40-4.64] | 5.97 [3.02-8.48] | 3.73 [3.05-7.73] | 4.46 [3.85-4.63] |
|  | 11^th^ gestational month | 6.71 [5.32-8.30] | 3.20 [2.24-3.85] | 1.56 [0.86-3.55] | 3.24 [2.23-4.59] | 4.09 [3.89-4.57] |
|  | 1 day *postpartum* | 4.81 [3.06-5.68] | 1.40 [1.13-1.57] | 1.84 [1.16-4.74] | 1.51 [1.15-2.09] | 1.16 [0.70-1.38] |
|  | 5 or 6 months *postpartum* | 1.03 [0.79-1.75] | 1.07 [0.81-1.34] | 1.20 [0.46-3.34] | 1.22 [0.65-1.57] | 0.99 [0.69-1.16] |

Values are presented as median [quartile 1-quartile 3]. P-P: Pony in Pony, P-D: Pony in Draft, S-P: Saddlebred in Pony, S-S: Saddlebred in Saddlebred, S-D: Saddlebred in Draft.
