# Supplementary material for: Enhanced or Reduced Fetal Growth Induced by Embryo Transfer into Smaller or Larger Breeds Alters Post-Natal Growth and Metabolism in Pre-Weaning Horses
Source: PLoS One. 2014 Jul 9;9(7):e102044. doi: 10.1371/journal.pone.0102044 (PMC4090198; doi:10.1371/journal.pone.0102044)
Supplement: Table S3 — Foals' parameters measured in the five groups. (DOC) [file pone.0102044.s003.doc]

**Table S3**. Foals’ parameters measured in the five groups.

|  | | P-P | P-D | S-P | S-S | S-D |
| --- | --- | --- | --- | --- | --- | --- |
| Body weight (kg) | day 0 | 25.5 [22.5-32.0] | 40.1 [33.6-40.9] | 31.0 [28.0-41.5] | 49.4 [43.9-55.4] | 53.8 [50.3-59.8] |
|  | day 180 | 135.0 [106.5-147.5] | 184.9 [170.8-201.0] | 152.5 [137.5-184.8] | 213.6 [195.3-234.4] | 223.9 [215.8-248.0] |
| Weight gain (kg.d^-1^) | day 0 to 180 | 0.60 [0.46-0.67] | 0.82 [0.75-0.90] | 0.69 [0.60-0.80] | 0.92 [0.83-1.00] | 0.95 [0.91-1.05] |
| Plasma IGF-1 (ng.mL^-1^) | day 0 | 233.7 [221.8-301.5] | 248.4 [236.5-289.4] | 198.3 [152.4-383.4] | 168.7 [145.7-200.1] | 174.7 [155.4-197.1] |
|  | day 3 | 388.6 [313.1-448.8] | 489.9 [393.6-572.3] | 191.6 [141.4-224.6] | 210.6 [165.5-255.0] | 216.0 [135.0-254.9] |
|  | day 90 | 777.2 [633.3-824.6] | 727.6 [674.1-824.2] | 287.8 [269.6-325.1] | 290.0 [236.2-378.9] | 421.7 [322.2-507.6] |
|  | day 180 | 554.7 [398.8-685.4] | 608.1 [550.9-744.4] | 321.2 [179.4-447.4] | 266.8 [199.1-309.9] | 202.5 [154.2-369.0] |
| Plasma T_3_  (ng.mL^-1^) | day 0 | 4.08 [3.71-5.04] | 3.96 [2.40-5.25] | 6.46 [5.60-8.10] | 5.87 [4.37-8.18] | 6.55 [4.58-10.49] |
|  | day 3 | 5.72 [4.15-6.30] | 2.55 [1.98-4.85] | 5.26 [4.40-6.10] | 2.75 [1.48-4.12] | 3.78 [1.92-6.04] |
|  | day 90 | 0.87 [0.75-1.33] | 0.59 [0.47-0.69] | 0.76 [0.56-1.11] | 0.54 [0.47-0.72] | 0.66 [0.60-0.74] |
|  | day 180 | 0.61 [0.51-0.75] | 0.32 [0.24-0.58] | 0.69 [0.51-1.01] | 0.41 [0.34-0.49] | 0.16 [0.14-0.23] |
| Plasma T_4_  (ng.mL^-1^) | day 0 | 392.3 [300.9-462.3] | 296.3 [209.0-317.8] | 484.5 [422.1-517.4] | 371.5 [303.7-414.0] | 268.4 [249.3-333.2] |
|  | day 3 | 219.2 [161.5-279.8] | 74.85 [57.62-89.93] | 238.0 [193.4-327.3] | 116.3 [85.8-203.8] | 99.22 [52.93-174.5] |
|  | day 90 | 26.81 [19.44-30.81] | 37.02 [21.25-46.85] | 36.60 [29.63-50.54] | 34.79 [24.94-45.93] | 34.81 [21.44-44.33] |
|  | day 180 | 27.31 [16.59-38.86] | 27.60 [22.18-32.52] | 23.68 [16.77-28.20] | 27.51 [19.33-32.77] | 22.77 [13.72-31.62] |
| Plasma T_3_ /T_4_ | day 0 | 0.011 [0.010-0.015] | 0.013 [0.008-0.022] | 0.014 [0.011-0.016] | 0.015 [0.011-0.026] | 0.027 [0.017-0.040] |
|  | day 3 | 0.025 [0.020-0.030] | 0.040 [0.028-0.067] | 0.023 [0.017-0.029] | 0.019 [0.014-0.029] | 0.045 [0.016-0.099] |
|  | day 90 | 0.035 [0.024-0.052] | 0.016 [0.011-0.029] | 0.019 [0.016-0.023] | 0.016 [0.011-0.025] | 0.017 [0.014-0.026] |
|  | day 180 | 0.022 [0.015-0.049] | 0.014 [0.009-0.020] | 0.038 [0.021-0.042] | 0.014 [0.012-0.020] | 0.007 [0.005-0.011] |
| Fasting glucose (mmol.L-1) | day3 | 6.56 [5.64-7.25] | 6.14 [4.26-7.18] | 7.36 [5.99-8.36] | 6.28 [5.24-6.56] | 5.72 [4.28-7.15] |
|  | day 30 | 8.72 [8.06-9.33] | 5.53 [4.90-6.06] | 8.58 [6.83-9.75] | 5.33 [4.72-5.89] | 5.95 [5.00-6.14] |
|  | day 90 | 6.72 [6.44-7.28] | 5.39 [5.04-6.06] | 7.03 [6.89-7.46] | 4.28 [3.86-5.13] | 5.12 [4.25-5.56] |
|  | day 140 | 5.67 [5.11-592] | 5.25 [4.51-5.74] | 5.17 [4.86-5.76] | 5.28 [4.67-5.86] | 4.20 [3.97-5.77] |
|  | day 180 | 5.72 [5.28-6.59] | 4.75 [4.06-5.36] | 6.81 [5.64-10.50] | 4.67 [3.86-5.31] | 3.81 [2.71-4.56] |
|  | day 200 | 5.67 [5.22-6.06] | 6.14 [4.92-6.54] | 5.92 [4.53-6.65] | 4.94 [4.50-5.75] | 4.47 [4.33-4.71] |
| Fasting insulin (ng.L-1) | day 3 | 0.94 [0.41-1.25] | 0.91 [0.51-1.32] | 0.43 [0.20-0.88] | 0.76 [0.10-1.34] | 0.91 [0.48-1.19] |
| Glucose AUC (mmol.min.L^-1^) | day 3 | 131.0 [102.8-142.8] | 162.1 [91.02-179.6] | 149.7 [106.7-204.7] | 160.0 [112.3-196.6] | 141.4 [101.1-180.5] |
| Insulin AUC (mmol.min.L^-1^) | day 3 | 14.03 [5.67-20.09] | 36.22 [16.52-61.46] | 7.47 [3.79-15.01] | 14.53 [7.28-33.27] | 16.19 [7.80-41.64] |
| Glucose metabolism rate (mmol/kg.min^-1^) | day 200 | 0.01260  [0.01081-0.01776] | 0.01163  [0.01054-0.01891] | 0.02468  [0.02044-0.03543] | 0.01953  [0.01409-0.02997] | 0.01622  [0.01297-0.01968] |

Values are presented as median [quartile 1-quartile 3]. P-P: Pony in Pony, P-D: Pony in Draft, S-P: Saddlebred in Pony, S-S: Saddlebred in Saddlebred, S-D: Saddlebred in Draft.
